# CRISPR/Cas9-Mediated Targeted Mutagenesis of *GmAS1/2* Genes Alters Leaf Shape in Soybean

**DOI:** 10.3390/ijms26199657

**Published:** 2025-10-03

**Authors:** Juan Xu, Mengyue Pan, Yu Zhu, Peiguo Wang, Liwei Jiang, Dami Xu, Xinyang Wang, Limiao Chen, Wei Guo, Hongli Yang, Dong Cao

**Affiliations:** 1 Key Laboratory of Biology and Genetic Improvement of Oil Crops, Ministry of Agriculture and Rural Affairs, Oil Crops Research Institute, Chinese Academy of Agricultural Sciences, Wuhan 430062, China; 2 National Nanfan Research Institute of CAAS, Sanya 572024, China

**Keywords:** soybean, *GmAS1/2*, leaf shape, wrinkling and curling, transcriptome analysis

## Abstract

*ASYMMETRIC LEAVES1* (*AS1*) and *AS2* play essential roles in regulating leaf development in plants. However, their functional roles in soybean remain poorly understood. Here, we identified two members of the soybean *AS1* gene family, *GmAS1a* and *GmAS1c*, which exhibit high expression levels in stem and leaf tissues. Using the CRISPR/Cas9 system, we targeted four *GmAS1* and three *GmAS2* genes, generating mutant lines with distinct leaf development phenotypes, including wrinkling (refers to fine lines and creases on the leaf surface, like aged skin texture), curling (describes the inward or outward rolling of leaf edges, deviating from the typical flat shape), and narrow. We found that functional redundancy exists among the four *GmAS1* genes in soybean. *GmAS1* and *GmAS2* cooperatively regulate leaf curling, leaf crinkling phenotypes, and leaf width in soybean, with functional redundancy also observed between these two genes. Transcriptome sequencing analysis of *w3* mutant (*as1b as1c as1d as2a as2b as2c*) identified 1801 differentially expressed genes (DEGs), including 192 transcription factors (TFs). Gene ontology enrichment analysis revealed significant enrichment of DEGs in pathways associated with plant hormone biosynthesis and signal transduction. A detailed examination of the DEGs showed several genes involved in the development of leaf lateral organs, such as *KNOX* (*SHOOT MERISTEMLESS* (*STM*), *KNAT1*, *KNAT2,* and *KNAT6*), *LOB* (*LBD25*, *LBD30*), and *ARP5,* were down-regulated in *w3*/WT (wild-type) comparison. CRISPR/Cas9-mediated targeted mutagenesis of the *GmAS1/2* genes significantly impairs leaf development and polarity establishment in soybean, providing valuable germplasm resources and a theoretical framework for future studies on leaf morphogenesis.

## 1. Introduction

Soybean plant architecture is a crucial trait for high-yielding cultivation, significantly impacting crop yield. This trait encompasses podding habit, main stem node number, plant height, petiole angle, branch number, internode spacing, internode length, leaf type, and leaf size [1,2]. Previous research on soybean plants has focused on stem growth habit [1,2,3,4,5]. Leaf shape, orientation, and petiole length are important factors affecting crop canopy structure and photosynthetic efficiency [3]. Favorable spatial distribution of leaves for light absorption significantly enhances and improves light use efficiency.

Dicot leaves consist of stipules, petiole, and leaf blade, organized along the proximal-distal axis. The regulation of proximal-distal axis polarity is epigenetically managed by a perinuclear deterrent complex composed of reciprocal allelic *ASYMMETRIC LEAVES1* (*AS1*) and *AS2* [6]. In *Arabidopsis*, *AS1* and *AS2* contribute to adaxial-abaxial leaf morphology and serve an essential function in leaf polarity establishment and overall morphogenesis [6,7,8,9,10]. The formation of the dorsal-ventral axis polarity is crucial in the development of leaf shape. Leaf primordia arise from the shoot apical meristem (SAM), which consists of undifferentiated cells. In the initial stages of leaf development, cell proliferation and lateral expansion are essential for establishing dorsoventral patterning, which is crucial for forming flat and symmetrical leaves [11]. *AS1/AS2* genes encode transcription factors (TFs) that are critical for dorsal-ventral polarity in leaf development and can directly or indirectly interact with multiple proteins or miRNAs [12]. Adaxial (dorsal) polarity determinants include genes from three families: the ARP, MYB (*AS1*), LOB (*AS2*), and HD-ZIP III (*REVOLUTA, PHABULOSA, PHAVOLUTA*). Conversely, abaxial (ventral) polarity determinants include genes from two families: the AUXIN RESPONSE FACTOR (ARF) (*ARF3, ARF4*), and KANADI, including *KAN1, KAN2, KAN3,* and *KAN4* [13,14].

The *AS1* genes encode an R2-R3 MYB domain protein, which functions as a transcriptional repressor and is crucial in forming the proximal-distal structure during leaf growth, exhibiting proximal- or distal-specific expression in leaves [15,16]. *AS1* shares homology with the *PHANTASTICA* gene from *Antirrhinum majus L.* and the *ROUGH SHEATH2* gene from *Zea mays*. *AS1* mutations in *Arabidopsis thaliana* result in lobed, dissected leaves that exhibit a distinct asymmetrical morphology. The asymmetric localization of auxin response at the distal leaf tip happens before the start of visible asymmetric growth. In maize, a recessive mutation in the thick leaf sheath gene (*rs2*), a direct homolog of *PHAN* and *AS1*, disrupts cell growth and parietal ligule positioning, leading to the loss of leaf sheath boundaries and a semi-leafless phenotype [17].

The *AS2* genes encode a protein containing a cysteine-repeat sequence within its LOB domain, which is crucial for the development of symmetrically flattened leaf lamina [18]. AS2 functions in the plant cell nucleus to regulate specific genes involved in midrib development, leaf vein patterning, and the establishment of symmetrically flattened leaves. Earlier research has demonstrated that mutants lacking *AS2* function exhibit downward leaf curling and other severe morphological defects [19,20]. Both *as1* and *as2* deletion mutants display similar leaf abnormalities, including leaf surface ruffling, asymmetric growth, margin curling, deeply incised cauline leaves, and ectopic leaflet formation [15,18,21]. Additionally, AS2 is essential for defining the proximal-distal polarity of leaves by inhibiting the gene *ETTIN* (*ETT/ARF3*)*,* which determines the distal axis [11]. The AS1-AS2-ETT regulatory pathway is a key for modulating advancement through the cell cycle and cytokinin biosynthesis, thereby stabilizing leaf development in *Arabidopsis thaliana* [11]. Furthermore, proper spatial and quantitative regulation of *AS2* expression is essential for correct leaf axis patterning [22].

In addition, AS1 physically associates with AS2 to form a repressor complex that directly attaches to the promoters of *KNOTTED1-like homeobox* (*KNOX1*) genes, which are responsible for regulating leaf shape and promoting stem cell activity in various plants. Additionally, *AS1* and *AS2* regulate proximal and distal parts of leaves by directly inhibiting two key genes, *BREVIPEDICELLUS (BP)* and *KNAT2*, which are found in the peripheral regions of subhyphal tissues of leaf primordia [7,8,9]. Furthermore, *KNOX* genes must be repressed for the formation of well-defined lateral organs [23]. It has been demonstrated that *KNOX*-mediated suppression of the gibberellin (GA) pathway in *as2* and *as1* mutants leads to smaller leaves and delayed flowering; a variety of polymorphic abnormalities, including the appearance of short-petioled leaf phenotypes, can be attributed to the aberrant expression of *BP*, *KNOTTED-LIKE ARABIDOPSIS THALIANA2 (KNAT2)*, and *KNAT6* [24].

Beyond that*, AS* genes are also associated with disease resistance in plants. The AS1 as a positive regulator for an extracellular defense mechanism against bacterial pathogens that does not rely on salicylic acid (SA) [25]. In contrast, the expression of *AS2*, *ERECTA,* and *KNOX* genes did not influence plant disease resistance [25].

To date, the functions of *AS* genes have been extensively studied. However, the regulatory roles of *GmAS1/2* in soybeans remain poorly understood. In our previous study, we utilized the CRISPR/Cas9 system to generate mutations in *GmAS1/2* genes in soybean [26]. Guan et al. identification of soybean homologs of *Arabidopsis AS1/AS2* via the Phytozome database; design of six target sites for seven *GmAS1/2* genes using the CRISPR-P tool (Figure S1); construction of sgRNA expression cassettes driven by *Arabidopsis* Pol III promoters (via BsaI-HF digestion and T4 ligation); assembly of these cassettes into the pYLCRISPR/Cas9P35S-BS vector; Agrobacterium tumefaciens strain EHA105-mediated transformation; selection of positive plants using phosphinothricin (PPT); and validation of editing efficacy via CTAB-based genotyping (PCR-sequencing) and phenotyping. Following genetic transformation, we observed that simultaneous mutation of all three *GmAS1* orthologs (*GmAS1a*, *GmAS1b*, *GmAS1c*) and all *GmAS2* orthologs (resulting in the *Gmas-m1* mutant) led to leaf phenotypes characterized by abaxial curling and wrinkling, as well as petiole shortening [26]. In this study, we focus on characterizing the mutation types and associated phenotypes of these multi-mutants and perform transcriptome analysis to elucidate the transcriptional regulatory network governed by *GmAS1/2* in soybean. Our findings are expected to provide both theoretical insights and practical applications for soybean architecture breeding.

## 2. Results

### 2.1. Expression Pattern of GmAS1 and GmAS2 Family Genes in Soybean

**Figure 1:**
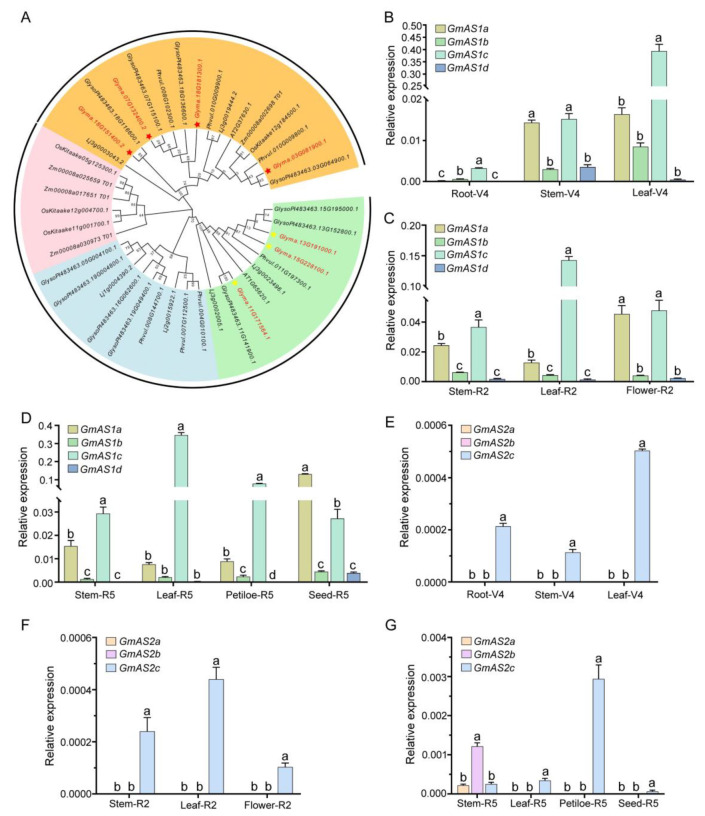
Phylogenetic analysis of *GmAS1/2* homologous genes and spatiotemporal expression patterns of *GmAS1/*2 homologous genes in Glycine max. (**A**) Using the Phytozome database, we systematically identified and analyzed *AS1/2* homologous gene sequences from *Arabidopsis thaliana*, *Glycine max*, *Phaseolus vulgaris, Glycine soja, Oryza sativa, Lotus japonicus, and Zea mays.* This phylogenetic tree revealed four well-supported evolutionary clades, indicated by orange, pink, blue, and green backgrounds, respectively. Among these, four *AS1* homologs in cultivated soybeans are marked with red asterisks, while three *AS2* homologs are denoted by yellow asterisks. (**B**–**D**) Relative expression levels of four *GmAS1* homologs across different tissues at the vegetative (V4), flowering (R2), and pod-filling (R5) stages. (**E**–**G**) Expression profiles of three *GmAS2* homologs at the corresponding developmental stages. One-way ANOVA was used for statistical analyses, followed by Tukey’s HSD test (*p* < 0.05). Statistically significant differences between groups are marked by different lowercase letters above the bars.

The *AS1/AS2* genes in plants are key transcription factors regulating leaf development. They play crucial roles in establishing the dorsal-ventral axis polarity of leaves and facilitating lateral organ formation. Evolutionary analysis reveals high conservation of *AS1/AS2* genes, particularly among dicotyledonous plants, where both structural and functional characteristics show significant conservation. In soybean (*Glycine max*), the genomic analysis identified four *AS1* orthologs (*GmAS1a*: *Glyma.07G132400*, *GmAS1b*: *Glyma.18G181300*, *GmAS1c*: *Glyma.03G081900*, *GmAS1d*: *Glyma.18G151400*) and three *AS2* orthologs (*GmAS2a*: *Glyma.13G191000*, *GmAS2b*: *Glyma.15G228100*, *GmAS2c*: *Glyma.11G171564*) (Figure 1A) [26].

To examine the expression patterns more thoroughly, we assessed the transcript levels of the *GmAS1* and *GmAS2* gene families across different soybean tissues. Their cDNA sequences were retrieved from the Phytozome database (https://phytozome-next.jgi.doe.gov/info/Gmax_Wm82_a4_v1, accessed on 14 July 2020), and specific primers were designed to assess their expression patterns across different tissues and developmental stages. The *GmAS1* and *GmAS2* gene families exhibited distinct expression profiles in various tissues (Figure 1). Among the orthologs of *GmAS1* and *GmAS2*, *GmAS1c* displayed the highest expression levels across the examined tissues at various stages of development, followed by *GmAS1a*. In comparison, the *GmAS2* genes exhibited generally low expression levels in soybean tissues. *GmAS2a* and *GmAS2b* demonstrated tissue-specific expression and were only detected in stem tissue at the R5 stage. Additionally, the transcription of *GmAS1* and *GmAS2* genes was nearly undetectable in root tissues, with the exception of *GmAS1c*.

### 2.2. CRISPR/Cas9-Mediated Mutagenesis of GmAS1 and GmAS2 Gene Families in Soybean

In our previous Chinese publication, we reported the generation of a sixfold mutant (with mutations in three members of the *GmAS1* and three members of the *GmAS2*) using the CRISPR/Cas9 system [26]. However, this work was presented merely as a technical report-focused primarily on the methodology of mutant creation, without in-depth analysis of the mutant’s phenotypic or genetic characteristics. Here, we provide a detailed characterization of these mutants and their related genetic variations.

**Figure 2:**
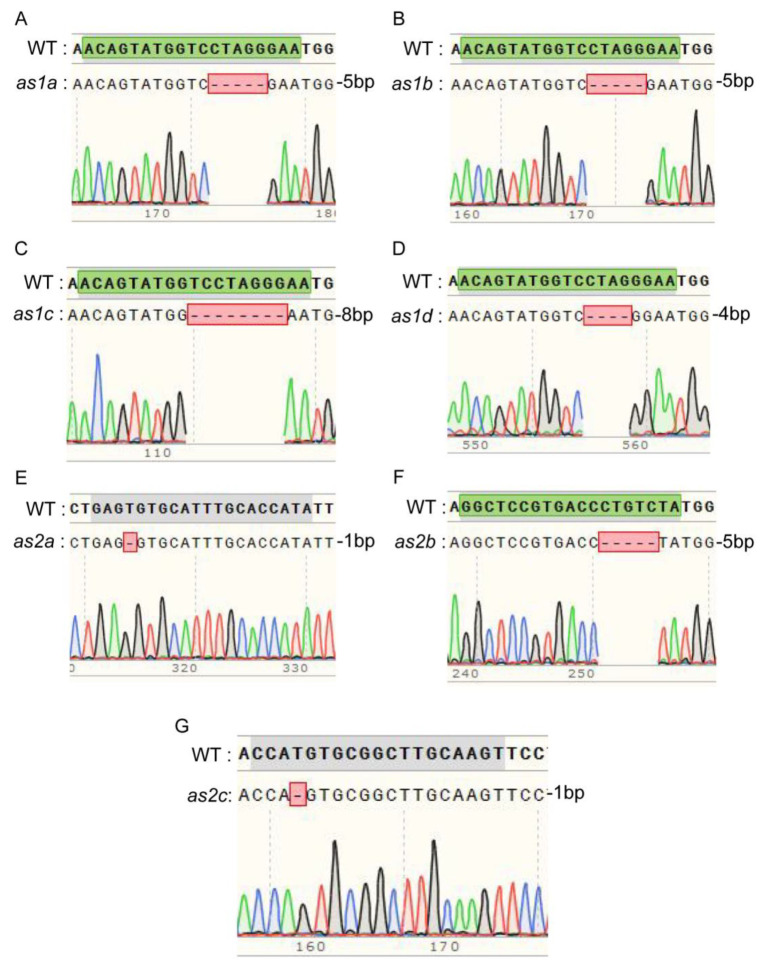
This figure displays the gene sequence alignment and Sanger sequencing electropherograms for the WT and various mutant lines of *GmAS1*/*GmAS2*, namely *as1a*, *as1b*, *as1c*, *as1d*, *as2a*, *as2b*, and *as2c*. Panels (**A**–**G**) highlight the sequence alterations in different *GmAS1* and *GmAS2* isoform mutants compared to the WT, which are marked by specific base deletions. These sequence polymorphisms are corroborated by distinct changes in the electropherograms, such as peak disruptions and positional shifts, thereby providing definitive molecular evidence for the sequence variations in *GmAS1* and *GmAS2*. This serves as a fundamental basis for elucidating the functional roles of these genes and their correlation with the phenotypic anomalies observed in the mutant lines.

We obtained three T0 transgenic lines (A, B, C) harboring the Bar gene expression cassette (Bar-positive). The seeds of 12 T1-generation plants were sown, and we found that these plants exhibited four distinct phenotypes compared with the wild-type (WT): normal (non-curled), moderately curled, distinctly curled, and extremely curled (Table S1). Sequencing analysis revealed that single mutants (*as2c*), double mutants (*as2b as2c* and *as1d as2c*), triple mutants (*as1d as2b as2c*), and quadruple mutants (*as1d as2a as2b as2c*)—which were heterozygous at the *as2a* locus—exhibited a WT-like phenotype (normal leaf morphology) compared to the WT. In comparison, the sixfold mutant exhibited extremely curled leaves relative to WT (Table S1). Notably, we obtained two transgenic free mutants, named *w1* (*as1d as2b as2c*) and *w2* (*as1d as2a as2b as2c*) from T1-generation (Figure 2, Table S1). To facilitate subsequent phenotypic investigation, we screened three additional transgenic-free homozygous mutants from the T2 generation, including one double mutant (*as2b as2c*), a new triple mutant (*as1a as2b as2c*), and a new sixfold mutant (*w3*: *as1b as1c as1d as2a as2b as2c*).

### 2.3. Phenotypic Characterization and Analysis 

**Figure 3:**
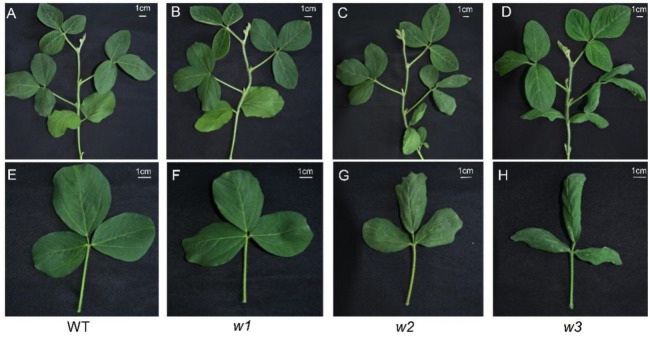
Phenotype of three transgene-free mutants *w1* (*as1d as2b as2c*), *w2* (*as1d as2a as2b as2c*), and *w3* (*as1b as1c as1d as2a as2b as2c*). (**A**–**D**) Represent pictures of the whole plants of WT (**A**), *w1* (**B**), *w2* (**C**), and *w3* (**D**) at the V3 stage, respectively. (**E**–**H**) correspond to the first compound leaf of the WT, *w1*, *w2*, and *w3*, respectively. The scale bar is shown in the top-right corner.

To quantitatively analyze phenotypic variation, nine individual plants were randomly selected from WT soybean plants and three mutant types (*w1*–*w3*) at the V3 stage. The width and length of simple leaves, as well as the central leaflets of the three compound leaves, were measured (Figures S2–S5). Additionally, the length/width ratio, as well as the ratios of length and width of leaves exhibiting phenotypic changes under artificially flattened and naturally crinkled conditions, were calculated to assess the degree of curling and crinkling in the mutants. We observed that only the simple leaves and the first compound leaves exhibited significant crinkling and curling (Figure 3). The second compound leaves showed curling only in length, while leaves closer to the shoot apex tended to display a normal phenotype. Therefore, the detailed analysis focused on the phenotypic data of simple leaves and the first compound leaves.

The results indicated that there were no significant differences in any of the measured parameters between the *w1* mutant plants and the WT. For the simple leaves of the w2 mutant under artificially flattened conditions, neither the chord-arc ratio nor the length-to-width ratio showed significant differences compared to WT. However, the width-based chord-arc ratio of *w2* simple leaves was significantly higher, while both the length/width ratio in their naturally crinkled state and leaf width (Figure S3) were significantly lower. This led to a notable increase in transverse curling in *w2* simple leaves, giving them a visually slimmer appearance.

**Figure 4:**
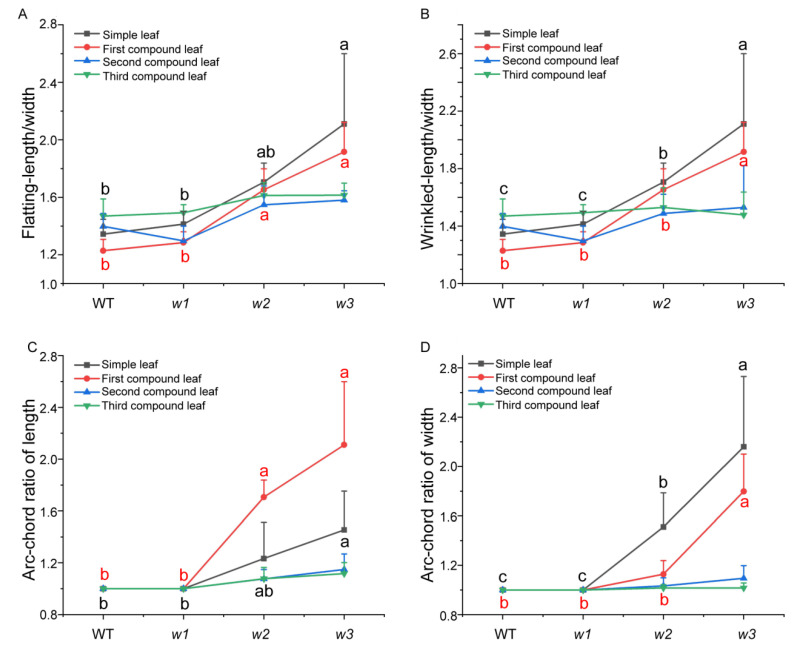
Morphological characteristic analysis of leaf wrinkling and curling degrees in various compound leaves of *GmAS1/2* mutants. Colored lines represent the parameter measurements for the simple leaf, and the middle leaflet of the first, second, and third compound leaves, respectively. (**A**) denotes the indicates the proportion of leaf length to width after manual flattening; (**B**) represents the ratio of length to width of leaves when they are manually flattened; (**C**) refers to the chord-arc ratio of leaf length, defined as the ratio between the leaf length when manually stretched and its length in the naturally wrinkled condition; (**D**) represents the chord-arc ratio of leaf width. Statistical annotations: Significant differences are shown by different lowercase letters (*p* < 0.05, ANOVA with Tukey’s test), while shared letters denote non-significant differences (*p* > 0.05). The figure uses black letters to denote significant differences in simple leaves between groups, and red letters to indicate significant differences in the middle leaflet of the first compound leaf among different phenotypes.

In contrast, for the first compound leaf of *w2* under natural crinkling, there were no significant differences in the width-based chord-arc ratio or length-to-width ratio between the mutant and WT (Figure 4). This suggests that the *w2* genotype does not influence the degree of transverse curling in the first compound leaf but does significantly enhance its longitudinal curling. Regarding the *w3* mutant, all measured phenotypic parameters were significantly greater than those of WT, showing pronounced increases in both transverse and longitudinal curling. As a result, the *w3* leaves appeared considerably slimmer than those of WT.

### 2.4. Comparative Analysis of WT and w3 Transcriptomes

**Figure 5:**
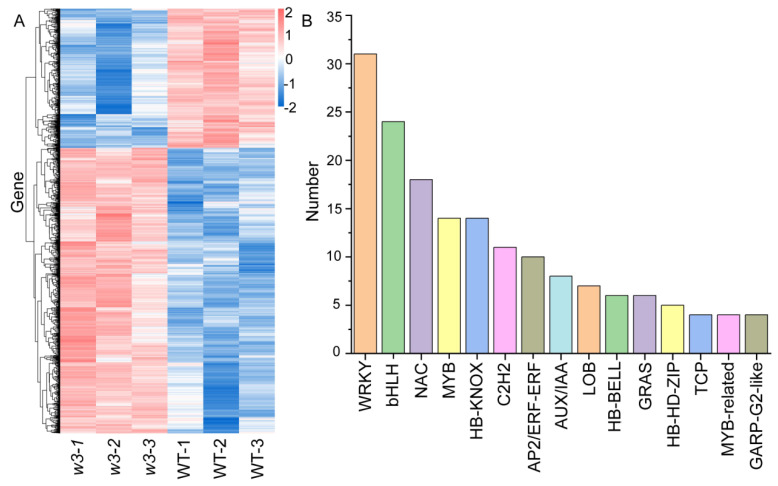
Differentially expressed genes (DEGs) profiling was conducted across the *w3* (*as1b as1c as1d as2a as2b as2c*) and WT. (**A**) Cluster analysis of DEGs for WT and *w3* is presented. *X*-axis: Sample name and sample clustering tree; *Y*-axis: Differentially expressed genes and gene clustering tree; color represents gene expression [log10 (FPKM + 0.000001)] in the corresponding sample. (**B**) This analysis covers transcription factors and transcriptional regulators identified by BMKCloud (www.biocloud.net, accessed on 13 September 2021) based on significantly differentially expressed gene predictions. The dataset includes 192 transcription factors and 13 transcriptional regulators. The figure emphasizes the transcription factor families that contain a significant number of members. Notably, the AUX/IAA family members are categorized as transcriptional regulators. Detailed counts of the remaining specific transcription factors and transcriptional regulators can be found in the Supplementary Materials.

To explore the molecular mechanisms through which *GmAS1* and *GmAS2* control leaf development, we performed RNA sequencing (RNA-seq) analysis on WT and *w3* (*as1b as1c as1d as2a as2b as2c*) mutant seedlings at the V3 growth stage. Selecting differentially expressed transcripts by applying a two-fold change (FC) threshold and a false discovery rate (FDR) cutoff of 0.05 for the *p*-value. Selecting differentially expressed transcripts by applying a two-fold change (FC) threshold and a false discovery rate (FDR) cutoff of 0.05 for the *p*-value, we identified 1801 differentially expressed genes (DEGs). In comparison to WT, 1213 genes were significantly increased in expression, while 588 genes were decreased in the *w3* mutant (Figure 5A, Supplementary Table S2).

TFs play pivotal roles in leaf development. Our analysis identified 192 DEGs annotated as TFs, belonging to 34 distinct TF families, including WRKY, bHLH, NAC, MYB, and HB-KNOX (Figure 5B, Supplementary Table S3). The WRKY family was the most common group of transcription factors, consisting of 31 differentially expressed genes, representing 15.7% of the total 192 differentially expressed transcription factors. This was succeeded by the bHLH family (24 DEGs, 12%), NAC family (18 DEGs, 9%), HB-KNOX family (14 DEGs, 7%), and MYB family (14 DEGs, 7%). Figure 5B shows the distribution of these TF families. *KNOX* genes, which stimulate stem cell activity, must be repressed to allow the formation of determinate lateral organs. Notably, 13 out of 14 *KNOX* genes were downregulated, including *KNAT1*, *KNAT2, KNAT6, and STM* in the *w3* (Supplementary Table S6). Comparative transcriptome analysis between WT and the *w3* identified several DEGs associated with leaf morphology, including significantly upregulated *GmAS1a.* In contrast, *GmAS2a*, *GmAS2b*, and *GmAS2c* were absent from the significant DEGs; however, six LOB domain-containing proteins exhibiting ~80% similarity to GmAS2, were identified. We detected 12 homologous genes containing the MYB DNA-binding domain (showing ~60% sequence similarity), with six genes demonstrating significant upregulation. The mean expression level of the genes that were upregulated was higher than that of the downregulated genes. In addition, transcriptome analysis also revealed significantly differentially expressed TFs, including HB-KNOX and LOB family members, which are involved in plant leaf development and morphogenesis. Thirty-nine DEGs associated with auxin were identified, and detailed information about these genes is provided in Supplementary Table S7.

### 2.5. Analysis of DEGs Using Gene Ontology (GO) and Kyoto Encyclopedia of Genes and Genomes (KEGG) Pathway Methods

**Figure 6:**
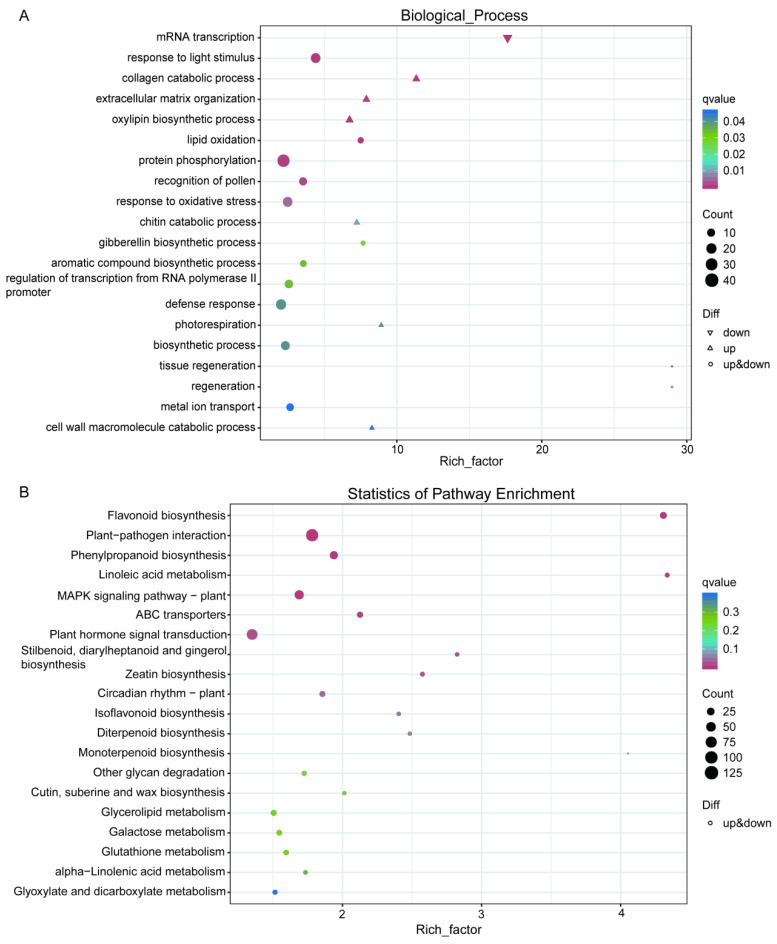
Analysis of differential gene expression: Enrichment of Gene Ontology (GO) terms and Kyoto Encyclopedia of Genes and Genomes (KEGG) pathways. (**A**) Bubble plot illustrating significantly enriched GO biological processes. (**B**) Bubble plot depicting significantly enriched KEGG metabolic pathways. In each panel, every dot corresponds to a particular GO term or KEGG pathway. The *Y*-axis represents functional terms or pathways, while the *X*-axis denotes the enrichment factor. The Rich factor is determined by dividing the proportion of DEGs associated with a specific term by the proportion of all genes linked to that term. A higher rich factor signifies a greater degree of enrichment for the term or pathway. Dot color corresponds to the q-value (i.e., adjusted *p*-value), with lower q-values signifying heightened significance and reliability of the enrichment signal. On the other hand, dot size reflects the count of genes enriched within each pathway, such that larger dots are indicative of a greater number of genes involved in the respective enrichment event.

To find the significantly enriched functional categories (GO terms) in *w3* compared to the WT, a gene set enrichment analysis was carried out. The Biological Process (BP) category, part of the top 20 enriched GO terms, included 793 DEGs, primarily associated with mRNA transcription, oxylipin biosynthesis, lipid oxidation, and response to light stimuli (Figure 6A, Supplementary Table S4). The most enriched term was protein phosphorylation. Among these processes, the extracellular matrix organization regulates cell wall formation, cell expansion, and mechanical strength. Gibberellin biosynthesis promotes stem elongation and seed germination, while lignin biosynthesis enhances cell wall strength and influences xylem development [27]. Oxylip ins interact with other phytohormones, such as gibberellins, to regulate plant growth and developmental processes.

To elucidate the molecular regulatory network underlying leaf phenotypic variation, we performed KEGG pathway enrichment analysis targeting the set of significantly DEGs. The results revealed significant enrichment of 770 DEGs across multiple metabolic pathways (Figure 6B, Supplementary Table S5), primarily including: secondary metabolic pathways such as flavonoid and phenylpropanoid biosynthesis, lipid metabolism (linoleic acid metabolism), stress response mechanisms (plant-pathogen interactions), and signal transduction mechanisms (MAPK signaling pathway). These mechanisms collectively regulate plant growth, development, and immune responses. Notably, the enrichment analysis also identified pathways closely associated with phytohormone regulation, including plant hormone signal transduction, zeatin (cytokinin) biosynthesis, and glutathione metabolism. These pathways participate in leaf morphogenesis by modulating the dynamic balance between cytokinins and auxins.

### 2.6. Confirmation of Chosen DEGs Through Quantitative Real-Time PCR (qRT-PCR)

**Figure 7:**
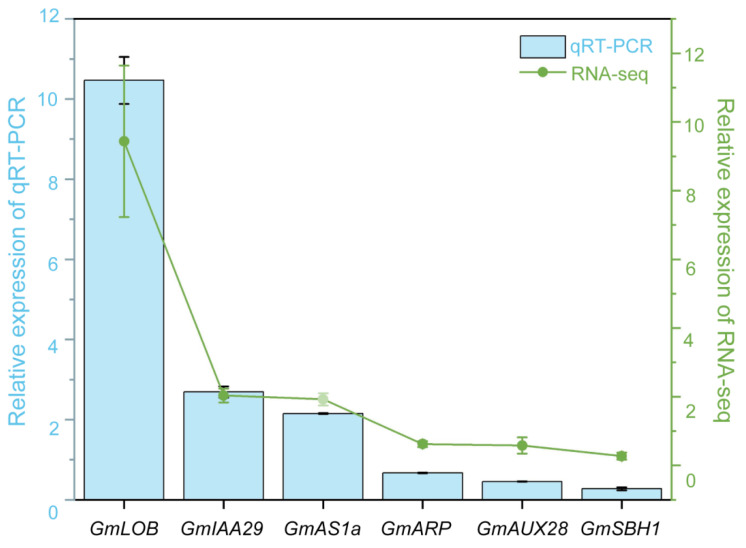
Validation of candidate gene expression in WT and *w3* plants, normalized to *GmActin* (*KP030799*). Relative expression levels determined by Quantitative real-time PCR (qRT-PCR) are presented in the line chart. *X*-axis: differentially expressed significant genes associated with *AS1/2* genes and leaf growth and development; *Y*-axis: On the left axis, the relative expression of DEGs—comparing the experimental group to the control group—is presented, with these values measured using qRT-PCR. The right axis denotes the relative expression levels based on the FPKM values of differentially expressed genes from the transcriptome data.

To confirm the differential expression patterns identified through RNA sequencing (RNA-seq) analysis, we selected six significantly DEGs for validation using qRT-PCR. These genes were chosen based on a thorough review of relevant literature and integrated transcriptome data analysis, with selection criteria emphasizing two main factors: a strong connection to *GmAS1/2* function such as the *KNOX* family gene *GmSBH1* (*Glyma.14G047000*), a key regulator of leaf morphogenesis suppressed by *GmAS1/2* or involvement in pathways related to leaf development (including phytohormone signal transduction and cell wall organization). The selected genes were *GmSBH1* (*KNOX* family), *GmIAA29* (*Glyma.17G165500*, AUX-related), *GmARP* (*Glyma.09G193000*), *GmAUX28* (*Glyma.19G161100*), and *GmLOB* (*Glyma.01G183900*, LOB-related). The qRT-PCR results closely matched the RNA-seq data regarding gene expression patterns (Figure 7).

## 3. Discussion 

To identify the function of the *GmAS1* and *GmAS2* genes in soybeans, multiple mutant lines were generated using a CRISPR/Cas9-mediated system. Through continuous multi-generation culture, we obtained mutant materials with different degrees of leaf wrinkling and curling. DNA sequencing confirmed multiple mutations in plants showing different degrees of leaf wrinkling. By comparing the mutant genotypes between the non-curled type (*as2c*; *as1d as2c*; *as2b as2c*; *as1d as2b as2c*; *as1a as2b as2c*) and the moderately curled type (*as1a as2a as2b as2c*; *as1d as2a as2b as2c*; *as1b as1d as2a as2b as2c*), it was found that the leaf curling phenotype occurred only when all three *GmAS2* genes were mutated. These results suggest that *GmAS2* is a key gene regulating soybean leaf shape, with functions that include increasing the degree of transverse and longitudinal leaf curling and reducing leaf width.

Further comparison of genotypes between moderately curled type and distinctly curled type (*as1a as1b as1d as2a as2b as2c*; *as1b as1c as1d as2a as2b as2c*; *as1c as1d as2a as2b as2c*) revealed that the phenotype of significant transverse and longitudinal curling (accompanied by wrinkling) in *w3* only appeared when two or more *GmAS1* genes were mutated, with one of the mutated genes being either *GmAS1a* or *GmAS1c*. This observation is consistent with the high expression levels of the two target genes during the V4, R2, and R5 stages, which further supports the presence of functional redundancy among the four *GmAS1* homologs in soybean. In conclusion, *GmAS1* and *GmAS2* cooperatively regulate leaf curling, crinkling phenotypes, and leaf width in soybean, with functional redundancy existing between these two genes. Consistent with prior findings in *Arabidopsis thaliana*, loss-of-function mutations in *AS2* have been shown to result in downward leaf curling and other severe morphological defects [19,20], while mutations in either *as1* or *as2* collectively induce leaf surface wrinkling, asymmetric growth, and marginal curling [15,18,21]. Separately, another study confirmed that *AS1* mutations in *Arabidopsis* lead to downward curling of rosette leaves and shortened petioles [28].

*LOB* genes constitute a plant-specific transcription factor family that plays pivotal roles in modulating plant morphological patterning and stress resilience [29]. Their expression is spatially restricted to the proximal adaxial-abaxial cell zones at the bases of lateral organs and lateral roots [30]. In this study, the leaf curling phenotype exhibited by mutants may be linked to six identified *LOB* domain-containing proteins, whose expression levels display notable variations. Prior research has established that in *Arabidopsis thaliana*, *AS1* and *AS2* assemble into a repressor complex. This complex directly interacts with the promoter regions of *KNOX* family genes—specifically *BP*, *KNAT2*, and *KNAT6* [23]—and in doing so, suppresses the transcriptional activity of *KNAT1*, *KNAT2*, and *KNAT6* [16]. In *as1* and *as2* mutant backgrounds, *KNOX*-driven inhibition of the gibberellin (GA) signaling pathway leads to diminished leaf dimensions and delayed floral induction [24]. This aligns with the outcomes of the current work: 13 *KNOX* genes (encompassing *KNAT1*, *KNAT2*, *KNAT6*, and *STM*) exhibited downregulated expression in soybean, a pattern that coincided with the occurrence of leaf crinkling and curling in the species. Mary et al. identified a negative feedback regulatory circuit between *AS1* and *KNOX* genes expressed in the shoot apical meristem of *Arabidopsis* [20]. Specifically, the *KNOX* gene *STM* exerts a negative regulatory effect on *AS1*, which then reciprocally represses other *KNOX* genes, including *KNAT1* and *KNAT2. AS2* functions at an equivalent regulatory tier to *AS1* within this network [20]. This regulatory framework may account for the marked upregulation of the unedited *GmAS1a* gene observed in the *w3* mutant in our study. Such upregulation could, in turn, enhance the repression of downstream *KNOX* genes, ultimately contributing to the leaf crinkling and curling phenotypes observed in soybean. Moreover, aberrant expression of *BP*, *KNAT2*, and *KNAT6* gives rise to a range of phenotypic anomalies, such as leaves with shortened petioles [24]. It is plausible that *GmAS1c* and *GmAS1a* act in a coordinated manner to regulate the expression of the downstream *BP* gene, thereby influencing petiole development. Additionally, transcriptomic data revealed a significant reduction in *ARP5* gene expression, which correlates with the observed phenotypic characteristic of drastically decreased leaf length and width in soybean. This finding mirrors a prior investigation that reported reduced leaf size in *Arabidopsis arp5* mutants [31].

The regulation of leaf shape and structure is heavily dependent on the auxin signaling pathway [32]. Our transcriptomic analysis identified 39 auxin-related DEGs (Supplementary Table S7), with *IAA27* exhibiting significant downregulation in the phytohormone signal transduction pathway. Research has demonstrated that the overexpression of *Arabidopsis TrIAA27* significantly promotes leaf elongation [33], while the overexpression of blueberry *VcIAA27* in *Arabidopsis* induces characteristic downward-curling leaves [34]. In our transcriptomic data, *IAA27*, a gene that exhibited significant downregulation within the plant hormone signaling pathway-may also be involved in regulating leaf curling in soybean. Additionally, the *Gretchen Hagen 3* (*GH3*) family, which is one of the three primary auxin-responsive gene families in plants, plays a key role in maintaining internal phytohormone balance by catalyzing the attachment of amino acids to plant hormones such as salicylic acid (SA), jasmonic acid (JA), and indole-3-acetic acid (IAA) [35]. The overexpression of the sweet orange (*Citrus sinensis Osbeck*) gene *CsGH3.1/CsGH3.1L* leads to a reduction in leaf area and upward-curling phenotypes, which are accompanied by significantly decreased levels of free auxin [36]. Similarly, the overexpression of *OsGH3.2* results in typical auxin-deficient small-leaf phenotypes [37]. These results collectively demonstrate that the observed changes in auxin-related gene expression and leaf morphological variations in our study (Figure 3, Supplementary Table S7) are highly consistent with the well-characterized functions of auxin-responsive genes. Notably, evolutionarily conserved components of the auxin signaling network, including *IAA27* and *GH3* family genes, likely mediate the formation of reduced leaf area and curling phenotypes in soybeans through precise regulation of endogenous auxin homeostasis.

Plant YABBY TFs critically regulate leaf blade expansion, adaxial-abaxial polarity establishment, as well as phytohormone signaling and stress responses [38,39]. Both YABBYs and KANADIs function as key regulators of abaxial polarity, with *KANADIs* constitutively activating *YABBYs* expression. Furthermore, *KANADIs* (*KAN1* and *KAN2*) directly suppress *AS2* transcription through promoter binding, thereby promoting abaxial cell fate specification during leaf development [40,41,42,43]. In our results, the expression of *YABBY4* was significantly down-regulated (Supplementary Table S8), which may be involved in leaf polarity changes.

## 4. Materials and Methods

### 4.1. Plant Cultivation and Growth Conditions

The soybean cultivar Tianlong No.1 (WT) and the corresponding edited mutants were cultivated in the greenhouse of the Institute of Oil Crops Research, maintained at 25 °C with a photoperiod of 14 h of light and 10 h of darkness. Additionally, they were grown at the Seed Industry Innovation Center and the National Nanfan Research Institute of CAAS, where the temperature was kept at 27 °C with a 12 h light and 12 h dark cycle. Each plant was individually grown in pots with dimensions of 12.5 cm in diameter, 14 cm in width, and 22.9 cm in height, with two plants per pot. The potting medium consisted of a 2:1 soil-to-vermiculite ratio.

### 4.2. Bioinformatics Analysis and Phylogenetic Assessment

Sequences related to genes were obtained from the UniProt plant genome database (https://www.uniprot.org/id-mapping/, accessed on 11 April 2025). Protein sequences alignment and phylogenetic trees were both performed using MEGA Version 7.0.26. The history of evolutionary trees was determined using the Neighbor-Joining method [44], which employed 1000 bootstrap replications used to assess tree topology and reliability. Evolview (https://evolgenius.info//evolview-v2/#login, accessed on 23 June 2025) was used to visualize the phylogenetic tree.

### 4.3. qRT-PCR Analysis

To comprehensively analyze the expression patterns of the *GmAS1* and *GmAS2* genes, total RNA was isolated from various plant tissues and mutant samples utilizing the RNeasy Plant Mini Kit (Takara, Shiga, Japan). Samples of tissue were gathered, including roots, stems, and leaves during the V4 vegetative stage; leaves, flowers, and stems during the R2 reproductive stage; and leaves, petioles, and developing seeds during the R5 reproductive stage, along with V3 vegetative stage samples of leaves from mutant material. To eliminate genomic DNA contamination, total RNA was extracted and treated with DNase. Subsequently, 1 µg of RNA was subjected to reverse transcription using the HiScript II Reverse Transcriptase (Vazyme, Nanjing, China) to generate cDNA. The synthesized cDNA was then diluted to one-tenth of its initial concentration and used as a template for qRT-PCR. The qRT-PCR was performed using SYBR Green PCR Master Mix on a Bio-Rad 9600 Real-Time PCR System (Bio-Rad, Hercules, CA, USA). Also, the candidate genes identified from the transcriptome analysis were validated using the methods above. Total RNA was extracted from young leaves at the V3 stage of *w3* (*as1b as1c as1d as2a as2b as2c*), which was created by incorporating an *AS* mutant line previously developed in our lab.

The samples were composite samples obtained from multiple biological replicates (N = 3), each undergoing three technical replicates. Primer sequences for the target genes and the reference gene *GmActin* (*KP030799*) can be found in Supplementary Table S9. Melting curve analysis was used to confirm the amplification specificity of each primer pair, and the expression levels of the target genes were standardized against *GmActin*, known for its consistent expression across various tissues and growth conditions [45]. Quantitative analysis was performed using system software by the comparative ΔΔCT method [46].

### 4.4. Creation of CRISPR Mutants

The nucleotide sequence of the four *GmAS1* and three *GmAS2* genes were downloaded from Phytozome (https://phytozome-next.jgi.doe.gov/info/Gmax_Wm82 a4_v1, accessed on 14 July 2020). The target sequence adaptors were designed using the web tool CRISPR-P (http://cbi.hzau.edu.cn/crispr/, accessed on 7 March 2018). To target the *GmAS1/2* genes using the CRISPR/Cas9 system, we constructed six single-guide RNA (sgRNA) expression cassettes, with the target sites depicted schematically in Figure S1. The CRISPR/Cas9 plasmid containing these sgRNA cassettes was initially introduced into Agrobacterium tumefaciens strain EHA105. Soybean transformation was subsequently carried out according to the protocol detailed by Bao et al. (2015) [47]. For screening transformants from the T0 to T3 generations, glufosinate was applied at a concentration of 160 mg L^−1^, and the treatment was maintained until the first compound leaf emerged.

### 4.5. DNA Extraction and Mutation Screening of GmAS1 and 2 Mutant Soybean Lines

The mutants exhibited leaves with varying degrees of curling or wrinkling, and we categorized the mutants into three groups based on the wrinkling and curling severity of their first compound leaves: *w1* (WT phenotype), *w2* (mildly wrinkled), and *w3* (severely wrinkled and curled). Genomic DNA was extracted from the leaves of each independent plant spanning the T1 to T4 generations and subsequently employed for PCR amplification. Target loci were amplified using sequence-specific primers (Table S9) through polymerase chain reaction (PCR). The resulting products were then subjected to first-generation sequencing and bioinformatics analysis conducted by Tsingke Biotechnology Co., Ltd. (Beijing, China). The genotypes of successfully edited mutants were determined by examining sequence peaks and comparing them with reference sequences. Specifically, heterozygous mutants were identified by the presence of overlapping peaks near the target site, while homozygous mutants were verified through sequence alignment with the WT sequence. Selected individual plants that tested negative for herbicide resistance were successfully edited for seed propagation and subsequent experiments.

### 4.6. Phenotypic Assessment

For phenotypic analysis, WT and T5 generation mutant plants (*w1*: *as1d as2b as2c*; *w2*: *as1d as2a as2b as2c,* and *w3*: *as1b as1c as1d as2a as2b as2c*) were grown in individual pots (three plants per pot) filled with a soil-vermiculite mixture (2:1 *v*/*v*, 280 g per pot). The plants were cultivated in an artificial climate chamber under controlled conditions: a 12 h light/12 h dark photoperiod and a constant temperature of 28 °C. At the V3 growth stage, nine plants each from the WT and the three mutant lines (*w1*, *w2*, *w3*) were selected for phenotypic measurements. The length and width of two leaf types were measured using a flexible ruler: simple leaves and the middle leaflets of the first, second, and third compound leaves. Measurements were taken under two leaf conditions: fully expanded and curled. Corresponding ratios were then calculated based on the collected data.

### 4.7. RNA-Seq Based Transcriptome Analysis

WT and T5 generation *w3* mutant plants were grown in pots measuring 18.5 cm in height and 18.5 cm in top diameter, with two plants per pot. The plants were cultivated in an artificial climate chamber under controlled conditions: a 16 h light/18 h dark photoperiod and a constant temperature of 28 °C. The shoot apexes were harvested at the V3 vegetative stage. Three biological replicates per line were used in the analysis. RNA extraction from soybean plants was performed using the RNAprep Pure Plant Kit (Tiangen, Beijing, China) in accordance with the manufacturer’s protocol. The quantity and purity of the extracted RNA were assessed using a NanoDrop 2000 spectrophotometer (Thermo Fisher Scientific, Wilmington, DE, USA). The integrity of the RNA samples was evaluated with the RNA Nano 6000 Assay Kit on an Agilent Bioanalyzer 2100 system (Agilent Technologies, Santa Clara, CA, USA). Library preparation and RNA sequencing were carried out by Biomarker Technologies Co., Ltd. (Beijing, China). The RNA-seq dataset, assigned the accession number PRJNA1272664, has been deposited in the NCBI database and will be made publicly available since 1 July 2026. The sequencing libraries were generated using the Illumina NovaSeq platform, yielding 150-base-pair paired-end reads in accordance with the manufacturer’s protocols. The raw sequencing data were processed utilizing BMKCloud (www.biocloud.net, accessed on 13 September 2021), an online bioinformatics pipeline. For alignment and subsequent analyses, a publicly accessible soybean genome assembly from Phytozome (https://phytozome-next.jgi.doe.gov/info/Gmax_Wm82_a4_v1/, accessed on 13 May 2025) was employed as the reference. DEGs between the two samples were identified based on log fold changes exceeding 2 and a false discovery rate (FDR) below 0.05, as determined by DESeq2 and EDSeq analyses. Transcription factor (TF) prediction was executed through BMKCloud, which also facilitated GO functional enrichment analysis and KEGG pathway analysis of the DEGs. GO terms and metabolic pathways were deemed significantly enriched when the Bonferroni-corrected *p*-value was less than 0.05.

To validate the differential expression patterns obtained from RNA-seq analysis, we conducted qRT-PCR to measure the transcript levels of six chosen DEGs. Total RNA was extracted from the same batch as transcriptome sequencing. For reverse transcription, approximately 1 µg of total RNA was utilized with the HiScript 1st Strand cDNA Synthesis Kit (Vazyme, Nanjing, China). qRT-PCR was conducted following established protocols. The soybean *GmActin* gene served as the reference for normalizing expression data. Reactions were carried out on a CFX-Connect™ Real-Time PCR System (Bio-Rad, Hercules, CA, USA) using iTaq™ Universal SYBR Green Supermix. Primer sequences for qRT-PCR are listed in Table S9. Expression levels were determined using the 2^−ΔCT^ relative quantification method, with data obtained from three biological replicates to ensure statistical reliability.

### 4.8. Data Analysis Using Statistical Methods

In this study, unless otherwise specified, all data points were analyzed with a minimum of three biological replicates to ensure reproducibility. The statistical significance of the results was evaluated using standard error calculations and Student’s t-test. Significance levels were indicated by asterisks, with * representing *p* < 0.05 and ** representing *p* < 0.01. Additionally, distinct lowercase letters in graphical representations denote significant differences between groups. All data are presented as mean values, with error bars indicating standard deviation (SD).

## 5. Conclusions

Based on the research conducted by Guan et al. on adaxial leaf curvature and wrinkling in *GmAS1/2* soybean mutants, our team has undertaken further in-depth studies. In soybean, *GmAS1* and *GmAS2* exhibit functional parallels with their counterparts in *Arabidopsis thaliana*, jointly governing key genes (such as *KNOX* and *ARF5*) and their associated auxin signaling pathways to influence leaf polarity and area. Simultaneous mutations in multiple homologs of *GmAS1* and *GmAS2* result in distinct soybean leaf phenotypes, including crinkling, marginal curling, and reduced leaf size.

By examining the tissue-specific expression profiles, we posit that *GmAS1a* and *GmAS1c* serve as pivotal effector genes orchestrating leaf morphological alterations. Upon synthesizing our experimental findings with existing literature, we propose the following regulatory framework in soybean leaves: a mutation in *GmAS1c*, the most highly expressed *GmAS1* homolog, results in a substantial reduction in *STM* expression in the *w3* mutant. STM, in turn, exerts negative regulation on *GmAS1* and *GmAS2*. Concurrently, the pronounced upregulation of *GmAS1a* counteracts the downregulation of *KNOX* genes (*KNAT1*, *KNAT2*, *KNAT6*), thereby influencing leaf polarity establishment. Through the utilization of various homologous gene mutation combinations and meticulous phenotypic evaluations, we have validated that *GmAS1* and *GmAS2* act in concert to modulate leaf area and curling, with *GmAS2* predominantly mediating longitudinal leaf curling. Our analysis of mutation combinations associated with diverse leaf crinkling and curling phenotypes has unveiled significant functional redundancy among the *GmAS1* and *GmAS2* homologs.

In conclusion, the results of this study lay a solid foundation for future research into the regulatory mechanisms governing soybean leaf morphological development. In follow-up studies, we plan to further validate these findings and delve into the underlying mechanisms to achieve a more comprehensive understanding of the molecular regulatory functions of *GmAS1/2* homologs in soybean leaf morphology.

## Data Availability

The original contributions presented in this study are included in this article; further inquiries can be directed to the corresponding author.

## Supplementary Materials

The following supporting information can be downloaded at: https://www.mdpi.com/article/10.3390/ijms26199657/s1.
