# Supplementary material for: CRISPR/Cas9-Mediated Targeted Mutagenesis of GmAS1/2 Genes Alters Leaf Shape in Soybean
Source: Int J Mol Sci. 2025 Oct 3;26(19):9657. doi: 10.3390/ijms26199657 (PMC12524711; doi:10.3390/ijms26199657)
Supplement: Supplementary file 1 [file ijms-26-09657-s001.zip › Figure S1.pdf]

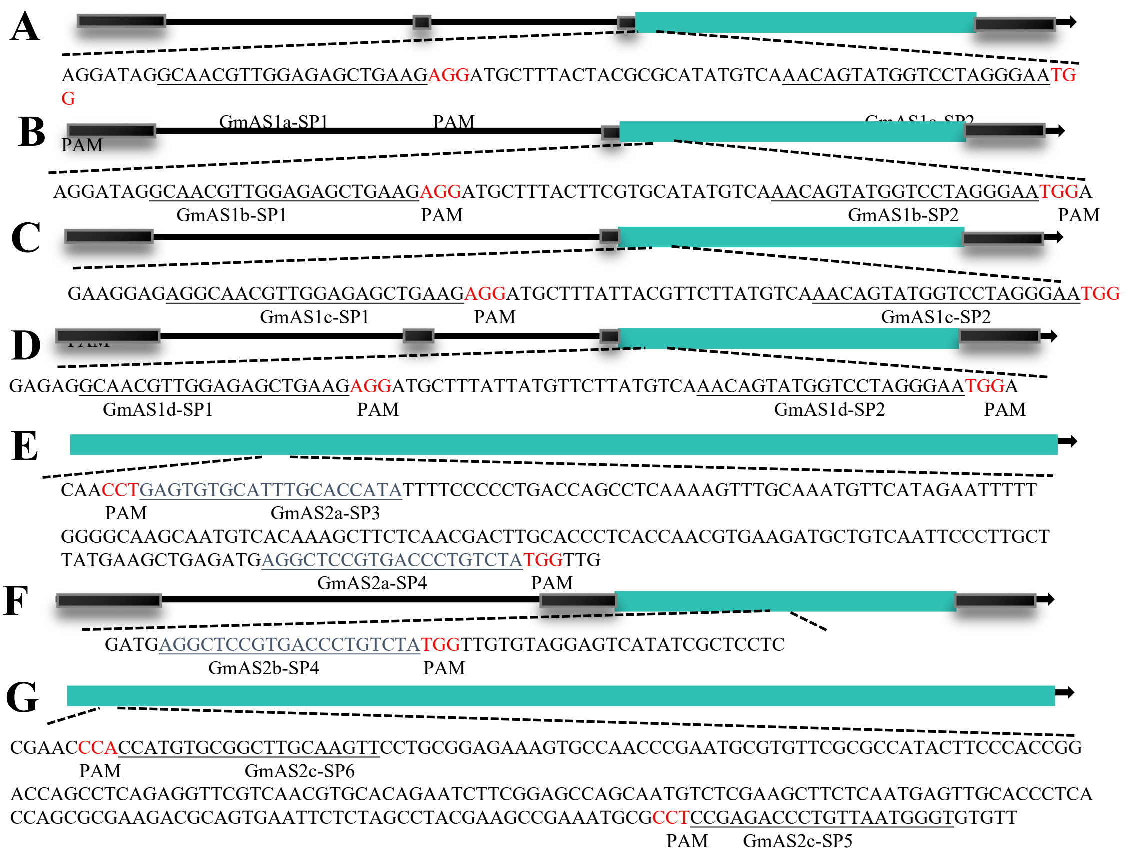

Figure S1. Schematic figure of target sites in seven AS gene

Note: A: Gene structure of GmAS1a with two target sites GmAS1a-SP1 and GmAS1a-SP2. B: Gene structure of GmAS1b with two target sites GmAS1b-SP1 and GmAS1b-SP2.C: Gene structure of GmAS1c with Two target site GmAS1c-SP1and GmAS1b-SP2. D: Gene structure of GmAS1d with two target sites GmAS1d-SP1 and AS1d-SP2.E: Gene structure of GmAS2a with two target sites GmAS2a-SP3 and AS2a-SP4. F: Gene structure of GmAS2b with one target sites AS2b-SP4. G:Gene structure of GmAS2c with two target sites GmAS2c-SP5 and AS2c-SP6. Nucleotides in red represent the protospacer adjacent motify (PAM). Nucleotides underlined indicate the target sites. Gray stripe, untranslated regions; orange stripe, exon; black line, intron.
