# Supplementary figures and images for: CRISPR/Cas9-Mediated Targeted Mutagenesis of GmAS1/2 Genes Alters Leaf Shape in Soybean
Source: Int J Mol Sci. 2025 Oct 3;26(19):9657. doi: 10.3390/ijms26199657 (PMC12524711; doi:10.3390/ijms26199657)

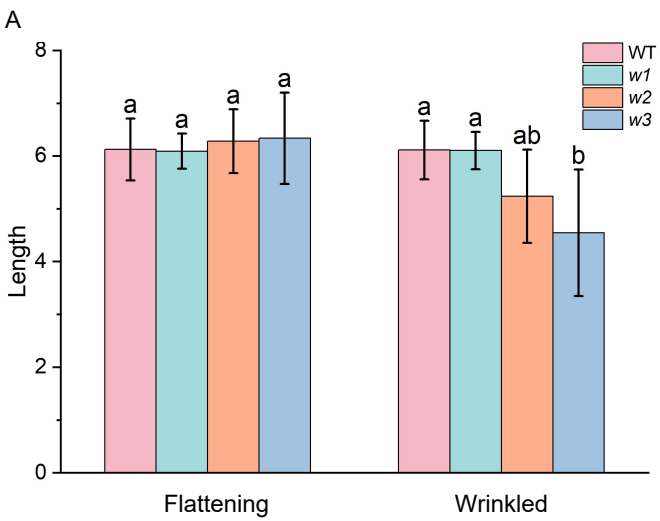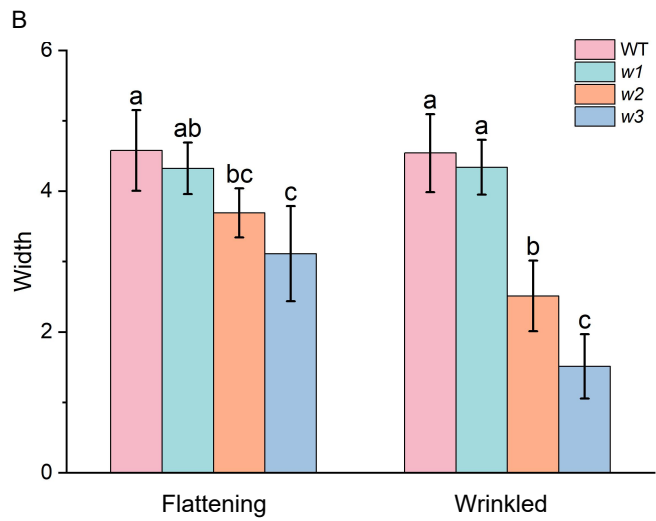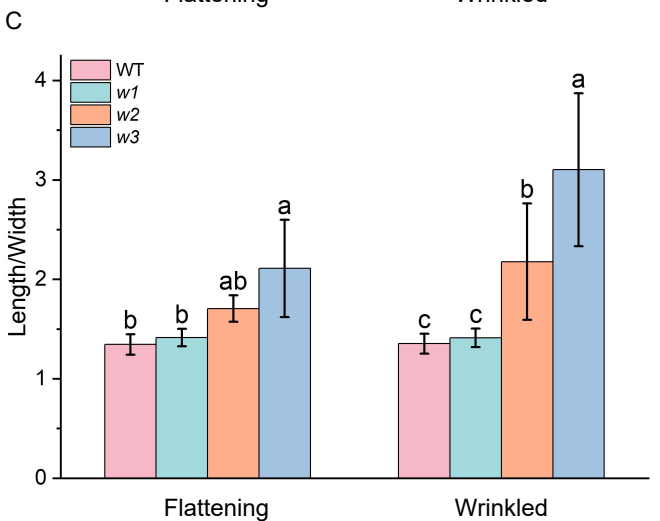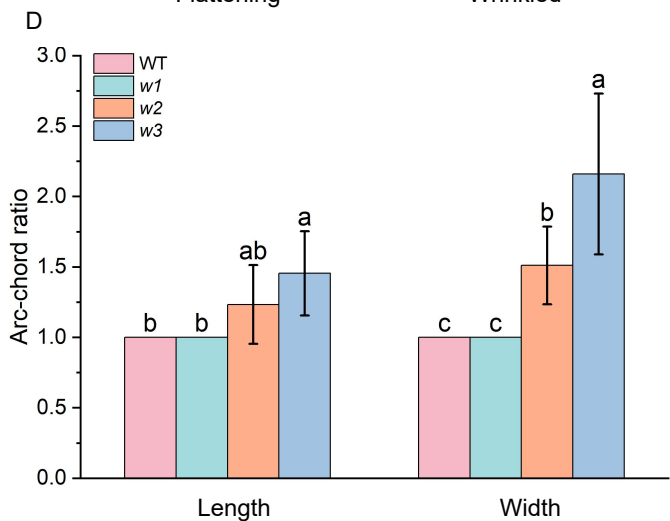

Supplement: Supplementary file 1 [file ijms-26-09657-s001.zip › Figure S2. Simple leaf.pdf]

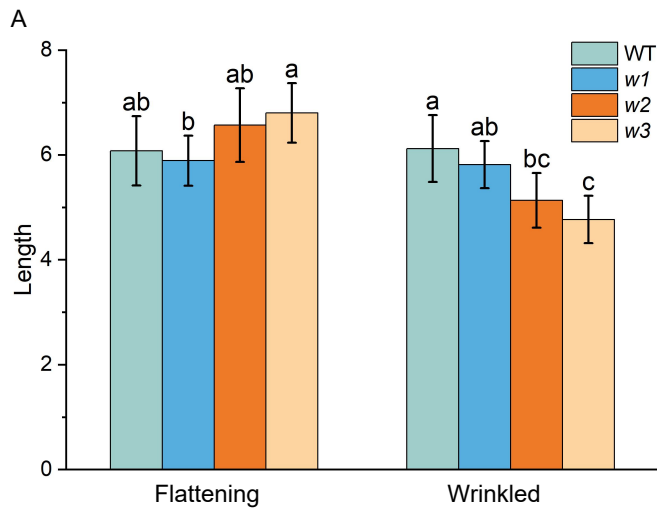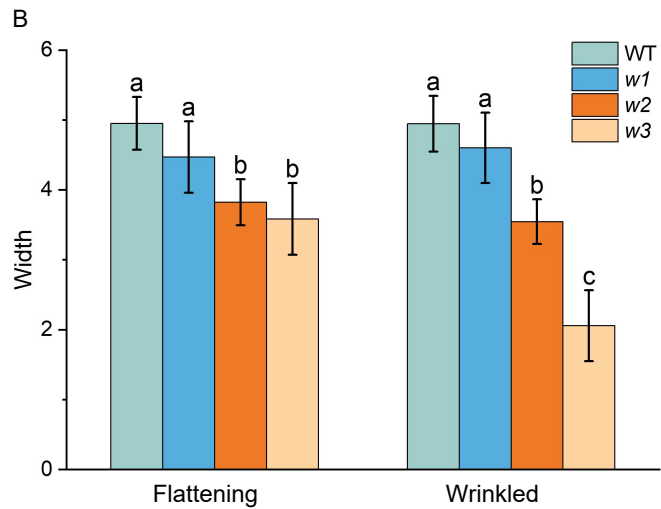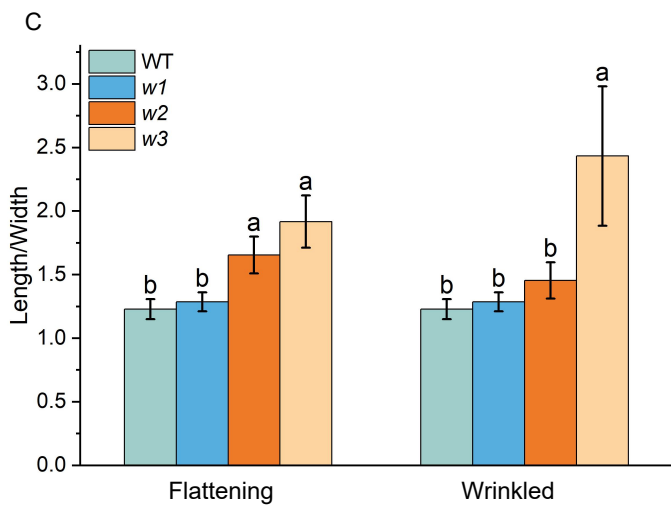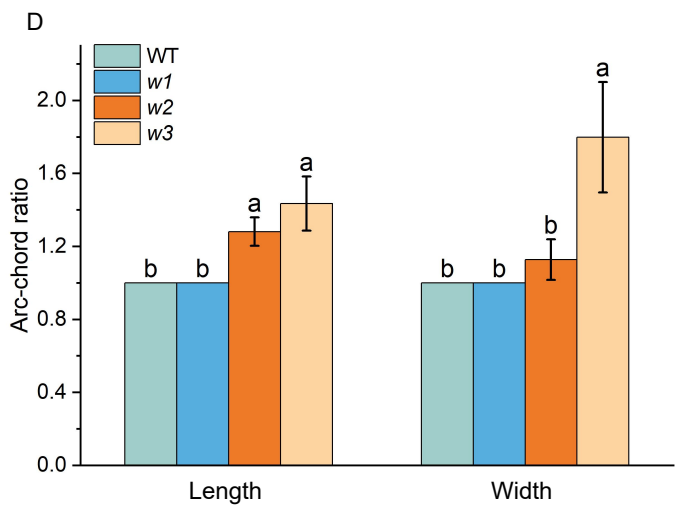

Supplement: Supplementary file 1 [file ijms-26-09657-s001.zip › Figure S3. First compound leaf.pdf]

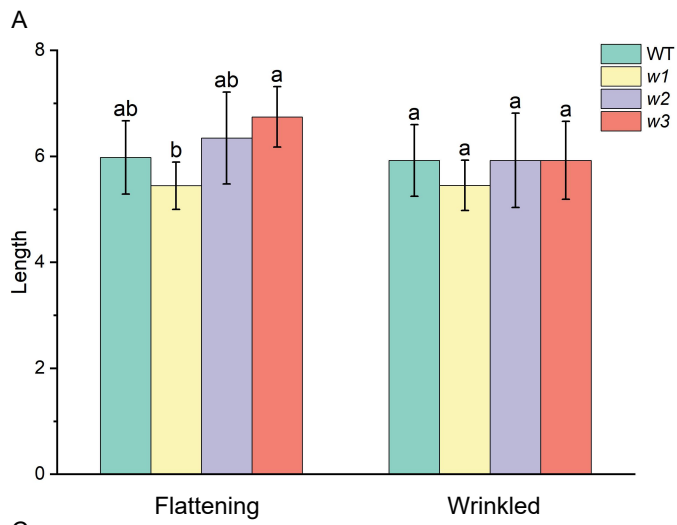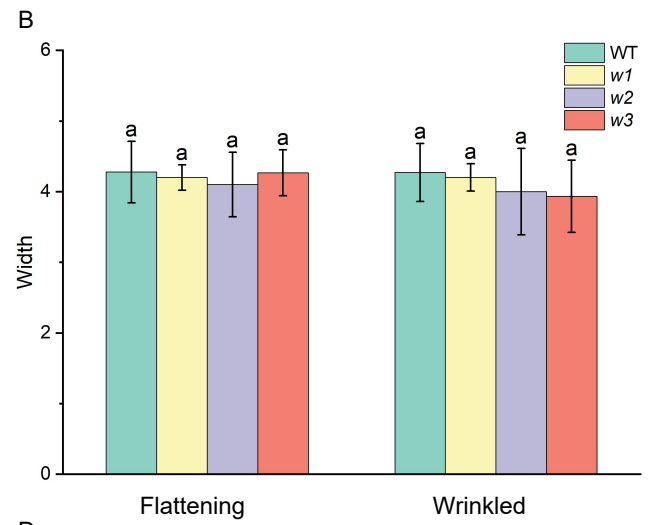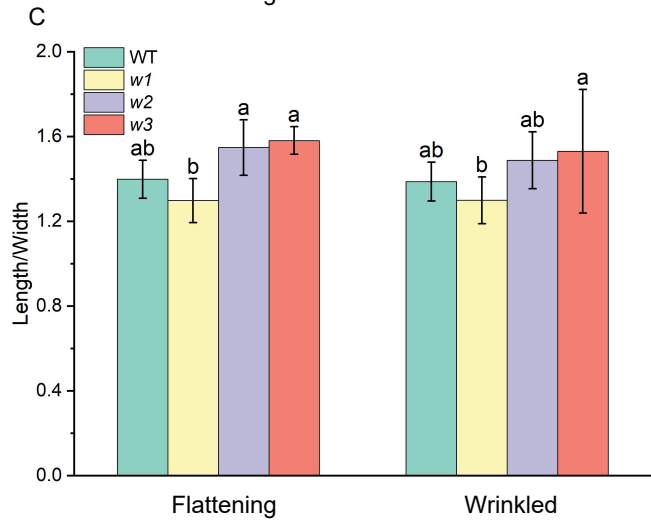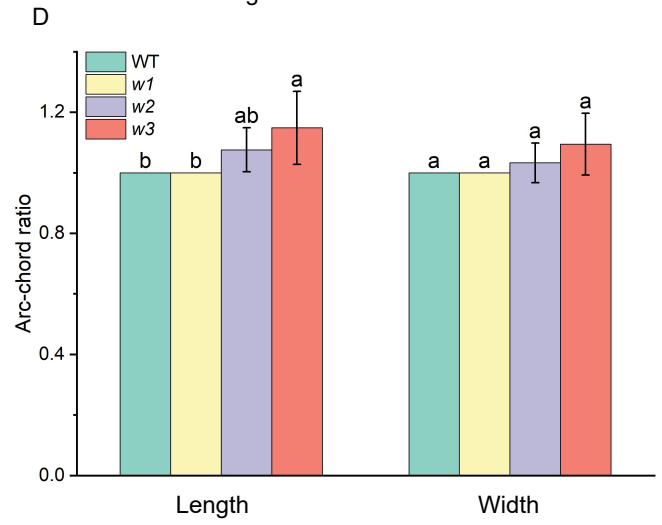

Supplement: Supplementary file 1 [file ijms-26-09657-s001.zip › Figure S4. Second compound leaf.pdf]

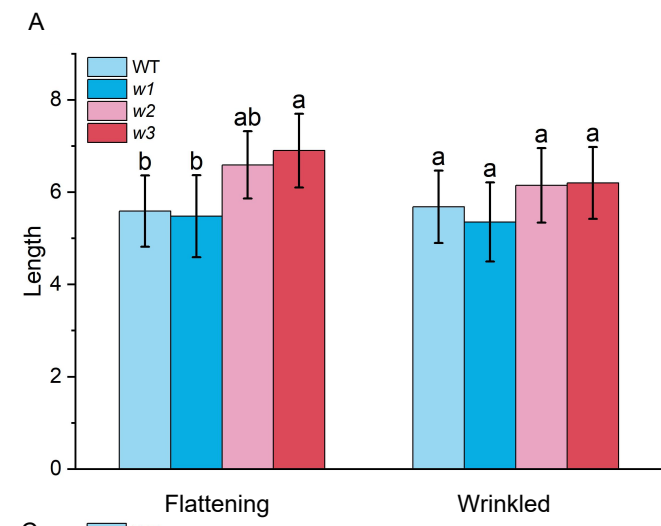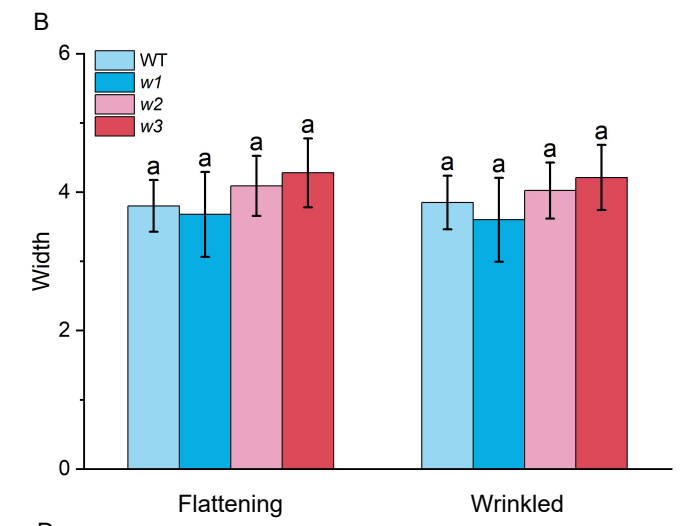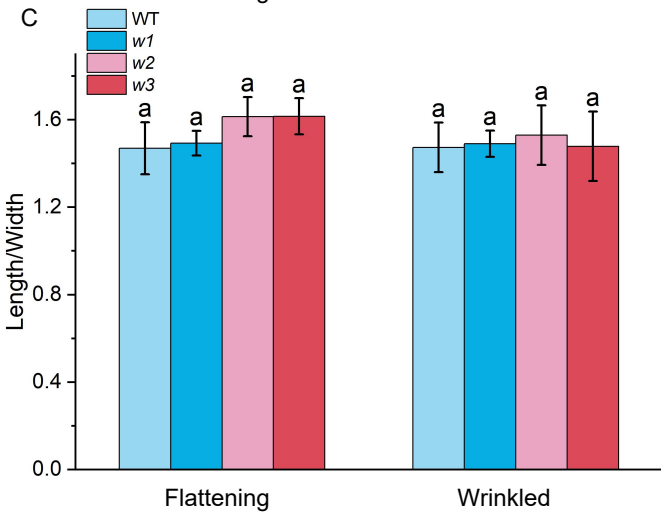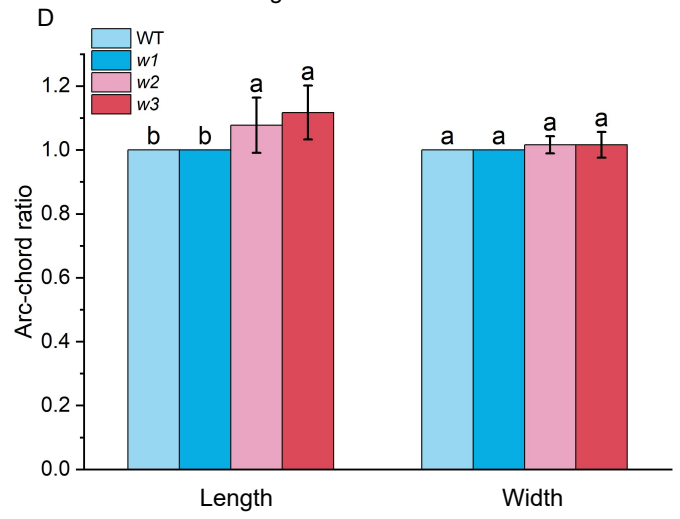

Supplement: Supplementary file 1 [file ijms-26-09657-s001.zip › Figure S5. Third compound leaf.pdf]
